# mRNA profiling reveals significant transcriptional differences between a multipotent progenitor and its differentiated sister

**DOI:** 10.1186/s12864-019-5821-z

**Published:** 2019-05-28

**Authors:** Laura D. Mathies, Surjyendu Ray, Kayla Lopez-Alvillar, Michelle N. Arbeitman, Andrew G. Davies, Jill C. Bettinger

**Affiliations:** 1 Department of Pharmacology and Toxicology, Virginia Commonwealth University, PO Box 980613, Richmond, VA 23298 USA; 2 Department of Biomedical Sciences, College of Medicine, Florida State University, Tallahassee, FL 32306 USA

**Keywords:** SGP, hmc, Multipotent progenitor, *C. elegans*, Transcriptome, Differential gene expression

## Abstract

**Background:**

The two *Caenorhabditis elegans* somatic gonadal precursors (SGPs) are multipotent progenitors that generate all somatic tissues of the adult reproductive system. The sister cells of the SGPs are two head mesodermal cells (hmcs); one hmc dies by programmed cell death and the other terminally differentiates. Thus, a single cell division gives rise to one multipotent progenitor and one differentiated cell with identical lineage histories. We compared the transcriptomes of SGPs and hmcs in order to learn the determinants of multipotency and differentiation in this lineage.

**Results:**

We generated a strain that expressed fluorescent markers specifically in SGPs (*ehn-3A::tdTomato*) and hmcs (*bgal-1::GFP*). We dissociated cells from animals after the SGP/hmc cell division, but before the SGPs had further divided, and subjected the dissociated cells to fluorescence-activated cell sorting to collect isolated SGPs and hmcs. We analyzed the transcriptomes of these cells and found that 5912 transcripts were significantly differentially expressed, with at least two-fold change in expression, between the two cell types. The hmc-biased genes were enriched with those that are characteristic of neurons. The SGP-biased genes were enriched with those indicative of cell proliferation and development. We assessed the validity of our differentially expressed genes by examining existing reporters for five of the 10 genes with the most significantly biased expression in SGPs and found that two showed expression in SGPs. For one reporter that did not show expression in SGPs, we generated a GFP knock-in using CRISPR/Cas9. This reporter, in the native genomic context, was expressed in SGPs.

**Conclusions:**

We found that the transcriptional profiles of SGPs and hmcs are strikingly different. The hmc-biased genes are enriched with those that encode synaptic transmission machinery, which strongly suggests that it has neuron-like signaling properties. In contrast, the SGP-biased genes are enriched with genes that encode factors involved in transcription and translation, as would be expected from a cell preparing to undergo proliferative divisions. Mediators of multipotency are likely to be among the genes differentially expressed in SGPs.

**Electronic supplementary material:**

The online version of this article (10.1186/s12864-019-5821-z) contains supplementary material, which is available to authorized users.

## Background

Embryonic stem cells are pluripotent; they can generate all cell types of the body, including cells from all three germ layers. Adult stem and progenitor cells can give rise to a more limited array of cell types and are therefore classified as multipotent. Although progress has been made in understanding the determinants of pluripotency [1], much less is known about the determinants of multipotency.

**Fig. 1:**
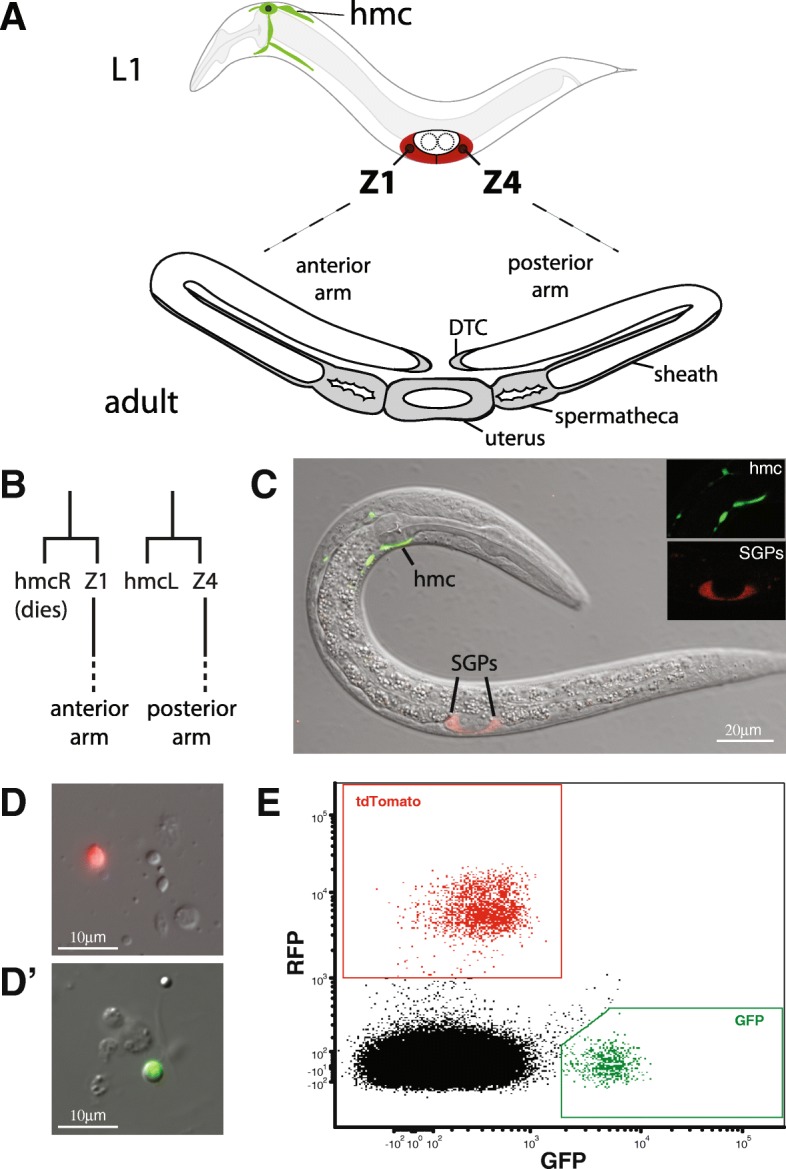
FACS sorting SGPs and hmcs from L1 larvae. (**a**) The SGPs (Z1 and Z4; red), and one hmc (green) are present in the first larval (L1) stage. The SGPs divide to produce support cells of the adult reproductive system, including distal tip cells (DTC), sheath, spermatheca, and uterus (grey). Each SGP produces one of the two gonadal arms: Z1 makes the anterior arm and Z4 makes the posterior arm. (**b**) Cell lineage leading to SGPs and hmcs. Precursor cells (not shown) divide asymmetrically to generate one SGP and one hmc. The hmcR cell dies by programmed cell death prior to the L1 stage. (**c**) Merged confocal differential interference and fluorescence microscopy image of an L1 stage worm with reporters expressed in the SGPs (*ehn-3::tdTomato,* red) and the hmc (*bgal-1::GFP,* green). Inset shows fluorescence images for each cell type. (**d**) Cell dissociates from L1 stage larvae showing individual cells expressing *ehn-3::tdTomato* (D, SGPs) and *bgal-1::GFP* (D’, hmcs). (**e**) FACS profile of dissociated cells from L1 larvae. GFP positive (green) and tdTomato positive cells (red) are outlined with boxes

The *C. elegans* somatic gonadal precursors (SGPs) are multipotent progenitors that generate all somatic cells of the adult reproductive system. The two SGPs, Z1 and Z4, are born during embryogenesis and they migrate to join the primordial germ cells (PGCs) to form the four-celled gonadal primordium [2]. SGPs remain quiescent until the first larval stage, when they go through two periods of cell division to produce all 143 cells of the mature hermaphrodite somatic gonad (Fig. 1a) [3]. The SGPs give rise to important regulatory cells, the distal tip cells (DTCs) and the anchor cell (AC), as well as complex multicellular tissues, including the sheath, spermatheca, and uterus (reviewed in [4]). The sisters of the SGPs are the two head mesodermal cells, hmcR and hmcL. hmcR undergoes programmed cell death late in embryogenesis and hmcL differentiates without further division as the single head mesodermal cell (Fig. 1b) [2]. The hmc cell extends cellular processes along the anterior-posterior and dorsal-ventral body axes to generate its distinctive H-shaped morphology [5]. The function of hmc remains unknown.

We previously reported that *hnd-1* and the SWI/SNF (SWItching defective/Sucrose Non-Fermenting) chromatin remodeling complex play roles in the SGP/hmc cell fate decision [6]. *hnd-1* encodes a bHLH transcription factor and the SWI/SNF chromatin remodeling complex regulates gene expression by altering chromatin structure. In animals carrying mutations in either of these transcriptional regulators, the SGPs usually express SGP-characteristic markers and migrate to form the gonadal primordium, but they can also express markers of the hmc cell fate and sometimes fail to develop into the tissues of the reproductive system [6]; this suggests that SGPs are often partially transformed into hmcs in these mutants. The incompletely penetrant phenotype of the mutations indicates that there are additional regulators of the SGP/hmc cell fate decision.

Here, we perform transcriptional profiling of isolated SGP and hmc cells to identify the gene expression differences underlying their distinctive cell fates. We find that the differentiated hmc cell expresses genes characteristic of neurons, suggesting that it has neuronal properties. In contrast, the SGP cells express genes involved in transcription and translation, which is consistent with the fact that they are poised to proliferate to generate the tissues of the somatic gonad.

## Methods

### Strains

*C. elegans* strains were cultured as described previously [7, 8]. All strains were grown at 20 °C unless otherwise specified and were derived from the Bristol strain N2. Strains were obtained from the *Caenorhabditis* Genetics Center or were generated as described below. The following alleles were used in this study and are described in *C. elegans II* [9], cited references, or this work:

*LGII: ttTi5605* [10].

*LGIII: unc-119(ed9)* [11], *ccIs4444* [*arg-1::GFP*] [12], *rdIs35* [*ehn-3A::tdTomato*] (this work).

*LGX: rdIs30* [*bgal-1::GFP*] (this work).

Reporter strains from the BC Gene expression consortium [13]:

BC15521 (*bgal-1::GFP*): *dpy-5(e907) I; sIs13743* [T19B10.3::GFP].

BC15463: *dpy-5(e907) I; sEx15463* [R151.2b::GFP].

BC12028 (*mrp-2::GFP*): *dpy-5(e907) I; sEx12028* [F57C12.4::GFP].

BC11529: *dpy-5(e907) I; sEx11529* [F48G7.10::GFP].

BC10183 (*asm-1::GFP*): *dpy-5(e907) I; sEx10183* [B0252.2::GFP].

BC11164 (*ahcy-1::GFP*): *dpy-5(e907) I; sEx11164* [K02F2.2::GFP].

BC11010 (*inx-9::GFP*): *dpy-5(e907) I; sEx11010* [ZK792.3::GFP].

### Reporter constructs

#### *ehn-3A::tdTomato* labels SGPs

We generated a single copy insertion of *ehn-3A::tdTomato* using the MosSCI technique [10]. The MosSCI repair plasmid was generated by excising *ehn-3A::tdTomato* from pRA351 [6] using ApaI and SpeI, blunting with T4 DNA polymerase, and cloning into pCFJ151 (Addgene #19330) that had been digested with XhoI and blunted with T4 DNA polymerase. The resulting plasmid (pRA528) was injected into EG4322 [*ttTi5605; unc-119(ed9)*] and inserted into the genome using MosSCI to generate *rdIs35*.

#### *bga-1::GFP* labels hmc

An hmc reporter strain (BC15521) was generated by the BC *C. elegans* Expression Consortium [13]. Although BC15521 was described as a chromosomal insertion, outcrossing revealed that it was a stable extrachromosomal array. We integrated the array containing the *bga-1::GFP* reporter into the genome by gamma irradiation to generate *rdIs30* and backcrossed it to N2 four times prior to use.

#### A genomic R151.2::GFP

We generated an R151.2::GFP reporter by CRISPR/Cas9 genome editing, as described previously [14]. The AP625–1 plasmid (Addgene #70051) containing eGFP coding sequence was modified to include a viral 2A “ribosome skipping” sequence N-terminal to eGFP [15]. We chose the T2A peptide because it produces nearly complete separation of flanking polypeptides in *C. elegans* [16]. AP625 was amplified with primers containing the T2A sequence and cloned using the Q5 site directed mutagenesis kit (NEB, Ipswich, MA). The resulting plasmid (pRA625) was used as a template for amplification with primers containing 35 bp overlap with R151.2; this PCR product serves as a repair template to insert T2A::GFP just upstream of the R151.2 stop codon. The guide RNA was selected using the Optimized CRISPR design tool (crispr.mit.edu) and purchased along with tracr RNA from IDT (Skokie, Illinois). The R151.2 guide will target Cas9 nuclease to cleave the R151.2 stop codon at the second position. We employed a co-conversion strategy using a *dpy-10* guide and repair oligo [17]. The RNA components (200 μM tracr, 20 μM *dpy-10* guide RNA, and 180 μM R151.2 guide RNA) were combined, heated to 95 °C for 5 min, and allowed to anneal at room temperature for 5 min. An injection mix, containing 1.5 μl of the annealed RNA mix, 1.8 μg repair template, 25 μg Cas9 protein (PNA Bio), and 5 pmol *dpy-10* repair oligo in a total volume of 10 μl, was assembled as described [14]. The mix was heated to 37 °C for 10 min and immediately injected into N2 worms. F1 roller worms were placed three to a plate and allowed to self-fertilize. Once the food was depleted, a portion of the population was washed off the plate and treated with proteinase K to produce a crude DNA prep. These DNA preps were screened using primers to R151.2 and GFP. Populations containing a PCR product of the correct size were singled to obtain homozygous R151.2::GFP. One R151.2::GFP homozygote was backcrossed two times to N2 to remove any off-target mutations introduced during the genome editing.

All primers used in this study are listed in Additional file 1: Table S1. Reporters were visualized using a Zeiss Axioskop II or Zeiss LSM710 microscope.

### Cell dissociation and FACS analysis

We generated a strain, RA587, containing *ehn-3A::tdTomato (rdIs35)* marking SGPs and *bgal-1::GFP* (*rdIs30)* marking the hmc, and used this strain to obtain populations of SGPs and hmcs. Five replicates were generated on different days. Cell dissociation was performed as previously described [18]. Briefly, 300,000–400,000 first larval stage (L1) worms were plated on 40–50 15 mm 8P plates seeded with NA22 bacteria and allowed to grow to adulthood [19]. Gravid adult worms were harvested from these plates and bleached to obtain populations of eggs. These eggs were hatched overnight in sterile M9 media on a rotating platform; animals hatched in the absence of food arrest development and become a synchronous early L1 stage population; at this stage of development, the SGPs and hmcs have been born and taken up their positions in the animal, but the SGPs have not begun to divide into differentiated tissues. The resulting L1 larvae were purified by sucrose flotation, washed twice with M9 media, and transferred to microcentrifuge tubes for dissociation. Worms were treated with SDS-DTT for 2 min, washed several times with M9, then treated with pronase (P8811; Sigma-Aldrich, St. Louis, MO) and mechanically disrupted for between 10 and 15 min. During the pronase step, samples were examined by fluorescence microscopy periodically to evaluate the dissociation. Cell dissociates were washed with L15 media, filtered through a 5 μm filter (MilliporeSigma, Burlington, MA), and resuspended in egg buffer. Cells were subjected to fluorescence-activated cell sorting (FACS) immediately.

Flow cytometry was performed at the Virginia Commonwealth University Flow Cytometry Shared Resource Core using an LSRFortessa-X20 (BD, Franklin Lakes, NJ) for initial analyses and a FACSAria II (BD, Franklin Lakes, NJ) with a 70 μm nozzle for cell sorting. Populations of SGPs (red fluorescence) and hmcs (green fluorescence) were obtained using FACS. We performed one test sort with DAPI to distinguish live from dead cells; DAPI can be taken up by the DNA of dead cells with disrupted membranes, but not by live cells. We observed no difference in the RNA quality of samples that were DAPI positive versus DAPI negative, therefore no DNA dye was used during the cell sorting. At least 20,000 cells were isolated for each cell type per replicate. Cells were sorted directly into Trizol (Ambion, Carlsbad, CA) and stored at − 80 °C until RNA preparation.

### RNA sequencing library preparation

Total RNA was isolated using the RNA Clean & Concentrator-5 kit (Zymo Research, Irvine, CA), with on-column DNase I digestion (Qiagen, Venlo, Netherlands). Test RNA preparations were performed with similar samples and yielded an average of 4.6 ng of total RNA per 10,000 cells as assessed by a Qubit 2.0 fluorometer (Invitrogen, Carlsbad, CA) and had RQI values ranging from 9.1 to 9.7 when analyzed using the Experion Automated Electrophoresis Station (Bio-Rad, Hercules, CA). Based on test preparations, we estimate that total RNA input was at least 10 ng for each sample. RNA sequencing libraries were prepared using the NEBNext Ultra II RNA Library Prep kit (NEB, Ipswich, MA) according to the manufacturer’s instructions, with 15 cycles of PCR amplification. The resulting libraries were quantitated by fluorometer and analyzed on a Bioanalyzer 2100 with the High Sensitivity DNA kit (Agilent, Santa Clara, CA). One library (hmc5) had low yield and showed evidence of significant primer dimers on the Bioanalyzer. This library was re-purified using AMPure XP beads (Beckman Coulter, Pasadena, CA) and amplified for four additional cycles as recommended by the manufacturer (NEB, Ipswich, MA).

### RNA sequencing and analysis

RNA sequencing was performed at the Genomic Services Lab at Hudson Alpha (https://gsl.hudsonalpha.org/index), using an Illumina HiSeq v4 2500 (Illumina, San Diego, CA). The libraries were sequenced as 50-base, paired-end reads, to an average read depth of 20 million reads per sample. We examined the raw RNA sequencing data using FastQC (https://www.bioinformatics.babraham.ac.uk/projects/fastqc/) for initial quality control purposes and found that some of the libraries contained Illumina adapter sequences. Trimmomatic version 0.36 [20] was used to remove Illumina adapters (ILLUMINACLIP parameters 2:30:10) and low quaility bases in leading and trailing ends, retaining sequences which were 36 bp or longer (LEADING:3 TRAILING:3 MINLEN:36). Sequences were aligned to the *C. elegans* genome (Ensembl genome assembly release WBcel325) using Tophat2 version 2.1.1 [21], with Bowtie2 version 2.3.3.1 as its underying alignment algorithm. The GTF option was used to provide Tophat with a set of gene model annotations and the following parameters were specified (max-multihits 1, mate-inner-dist 200, −I 18000 –I 40). We examined the data for quality, consistency, and overall sequence content using the RNA-Seq QC plot in SeqMonk (https://www.bioinformatics.babraham.ac.uk/projects/seqmonk/) and found that, with the exception of hmc5, the libraries contained mostly genic and exonic sequence with minimal rRNA contamination (Additional file 1: Table S2). Because the hmc5 library underwent additional rounds of amplification and showed significant ribosomal RNA contamination, we did not include this hmc replicate in subsequent analyses. Aligned reads were sorted and indexed using SAMtools [22]. Gene-based read counts were obtained using HTSeq version 0.6.1 [23], with the union overlap resolution mode and using the Caenorhabditis_elegans.WBcel235.86.gtf annotation file. Differential expression was determined using DESeq2 [24], and FPKM (Fragments Per Kilobase of Exon Per Million Fragments Mapped) values were obtained using Cufflinks version 2.2.1 [25]. Principle component analysis was performed on regularized log transformed data using the rlogTransformation and plotPCA functions in DESeq2 [24], to visualize the variance among our replicates and samples. Filtering based on FPKM was performed on the mean FPKM value for a given cell type. MA and volcano plots were generated from read counts using iDEP [26] with filtering to remove genes with fewer than 0.5 counts per million in at least four replicates. Overrepresentation of GO terms for the differentially expressed genes (DEGs) was determined using the statistical overrepresentation test in PANTHER [27–29]. Gene lists were compared to all *C. elegans* genes in PANTHER using the GO-slim Biological Process dataset and Fisher’s exact test with false discovery rate (FDR) correction.

## Results

### mRNA profiling of isolated SGPs and hmcs

In order to isolate SGPs and hmcs from the same animals, we generated a strain that expresses a red fluorescent protein in SGPs (*ehn-3A::tdTomato*) and a green fluorescent protein in hmcs (*bgal-1::GFP*). In first larval stage (L1) worms, these reporters are expressed exclusively in SGPs and hmcs (Fig. 1c). We synchronized populations of L1 larvae and dissociated SGPs and hmcs using published protocols for isolating larval cells from *C. elegans* [18, 30]. The larval dissociation yielded individual SGPs and hmcs (Fig. 1d-d‘), which, when analyzed by flow cytometry, showed distinct populations of red and green fluorescent cells (Fig. 1e). We isolated populations of SGPs and hmcs using fluorescence-activated cell sorting (FACS). Each L1 larva has two SGPs and one hmc, so the expected ratio of SGPs (red fluorescence) to hmcs (green fluorescence) is 2:1. Our individual sorting experiments varied in the ratio of SGPs to hmcs and they were generally skewed toward a higher than 2:1 ratio. The higher ratio of SGPs to hmcs may have occurred because the hmc is more difficult to dissociate as an intact cell from L1 larvae, owing to its elaborate cellular morphology, or the SGPs may be easier to dissociate due to their central location. We performed five independent cell isolations and obtained at least 20,000 cells of each type for each experiment.

**Fig. 2:**
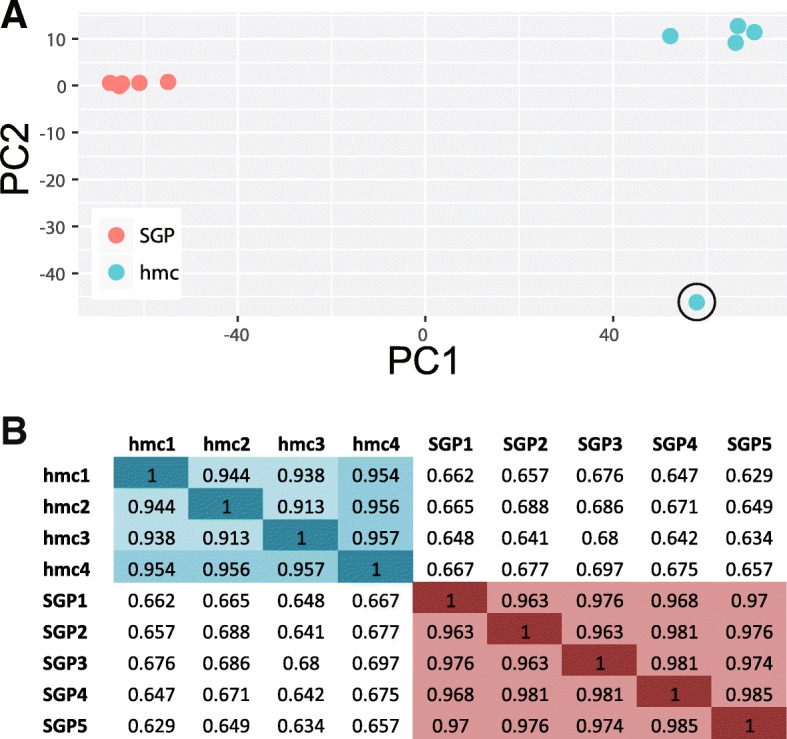
Principle component analysis of SGP and hmc gene expression. (**a**) Gene expression profiles plotted against the first two principle components (PC1 and PC2). The SGP and hmc replicates are most similar to one another. One hmc replicate (hmc5) had an expression profile that was significantly different from the other hmc replicates (circled); this sample was not used in subsequent analyses (see Methods). (**b**) Pearson’s correlation coefficients for each pairwise comparison. The SGP and hmc replicates show strong correlation within cell type

We assessed the correlation between biological replicates using principle component analysis and found that the SGP and hmc biological replicates clearly grouped together (Fig. 2a). The first two principle components accounted for 96% of the variance in the dataset, with principle component one (variation between sample types) accounting for 90% of the variance. One hmc replicate was significantly different than the other four replicates (Fig. 2a, circled). This sample required additional rounds of amplification during library preparation (see Methods) and contained significant rRNA contamination (Additional file 1: Table S2); it was therefore excluded from subsequent analyses. Pearson’s correlation coefficients ranged from 0.913 to 0.957 for the remaining hmc replicates and from 0.963 to 0.985 for the SGP replicates (Fig. 2b).

### SGPs and hmcs are transcriptionally different

**Fig. 3:**
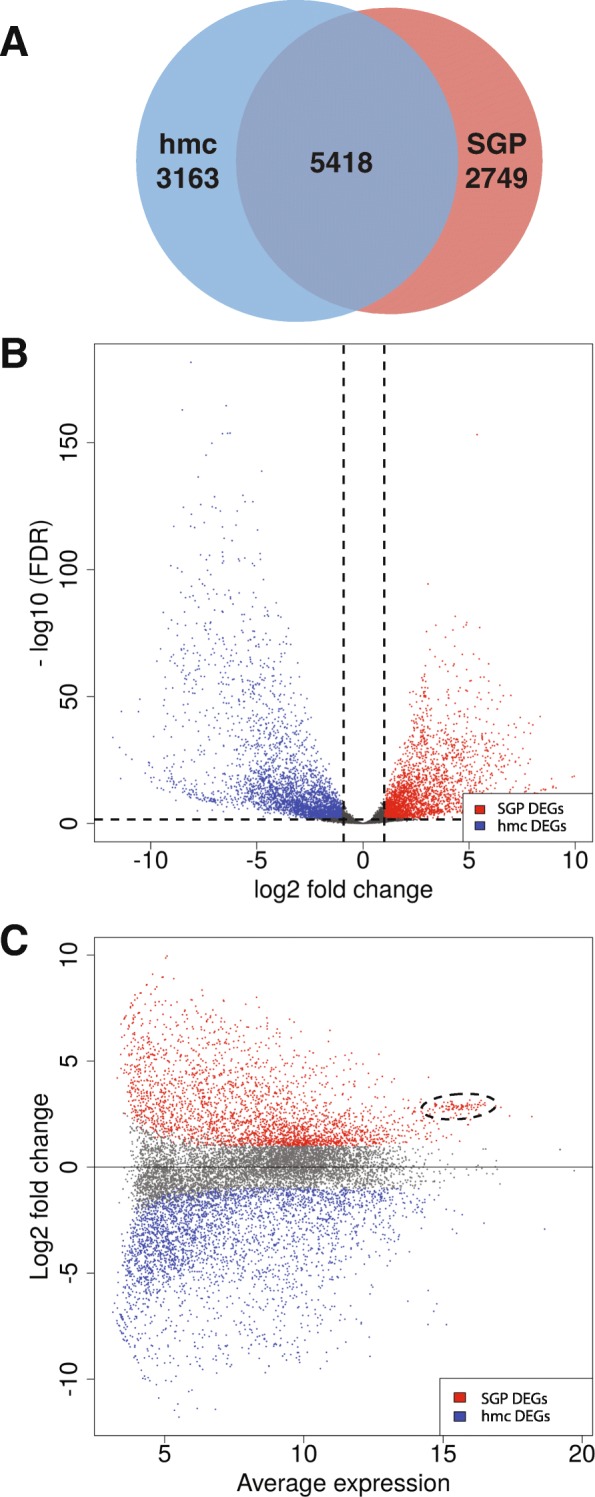
Analysis of differentially expressed genes in SGPs and hmcs. (**a**) In total, we detected transcripts from 11,330 genes (mean FPKM > 1). Differential gene expression analysis identified 5912 genes with differential expression between SGPs and hmcs (FDR ≤ 0.01, fold-change ≥2). Of these genes, 2749 have higher expression in SGPs and 3163 have higher expression in hmcs. 5418 genes show expression in at least one of the two cell types, but do not have significantly different expression between the two sample types. (**b**) Volcano plot shows genes that are differentially expressed in SGPs (red) and hmcs (blue). Dashed lines indicate the FDR and fold change cutoffs (FDR ≤ 0.1 and fold change ≥2). (**c**) MA plot showing genes that are differentially expressed in SGPs (red) and hmcs (blue). A cluster of genes has a high average level of expression and is differentially expressed in SGPs (dashed oval). This cluster includes genes involved in ribosomal biogenesis, such as ribosomal protein-encoding genes

In total, we detected transcripts from 11,330 genes (mean FPKM > 1; Additional file 2). We analyzed differential gene expression using DESeq2 [24] and found that 5912 genes were differentially expressed between SGPs and hmcs (FDR ≤ 0.01, fold-change ≥2) (Additional file 2). Similar numbers of genes were up- and down-regulated in SGPs when compared to hmcs (Fig. 3a); we observed higher expression in SGPs for 2749 genes (46.5%) and in hmcs for 3163 genes (53.5%). A volcano plot shows the wide distribution of differentially expressed genes (DEGs) (Fig. 3b).

**Fig. 4:**
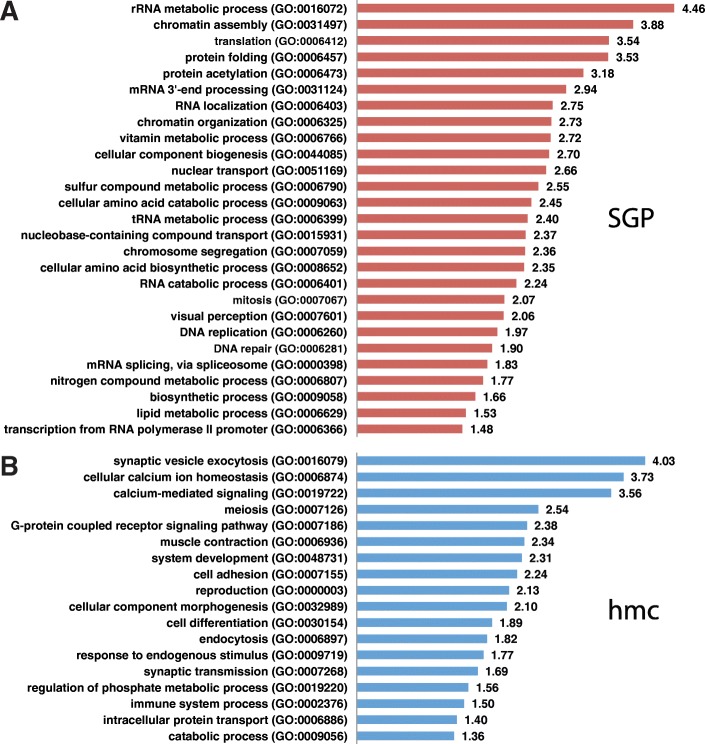
GO term overrepresentation analysis. PANTHER GO slim biological process terms enriched in the SGP (**a**) and hmc (**b**) DEGs. GO terms are plotted against the fold enrichment relative to the expected number of gene lists of these sizes

We found that gene ontology (GO) biological process terms associated with cell proliferation were highly overrepresented among the DEGs with SGP-biased expression (Fig. 4a; Additional file 3). For example, there were 4.5 times more genes associated with “rRNA metabolism” and 3.5 times more genes associated with “translation” than would be expected for a gene list of this size (FDR < 0.05). Genes associated with translation and ribosomal function, for example ribosomal protein-encoding (*rps* and *rpl*) genes, fall into a distinct cluster on the MA plot (Fig. 3c), showing some of the highest SGP-biased expression in this experiment. Also notable in the overrepresented GO terms for SGP-biased genes was “transcription from RNA polymerase II promoter”. Genes within this GO term category include several that encode transcription factors and chromatin regulators (Table 1; Additional file 3). Each of these GO terms is indicative of a cell that is preparing for cell division and subsequent development.

**Table 1:** Genes with GO term “transcription from RNA polymerase II promoter” are enriched in SGP DEGs

Gene^a^	Description
*C16C10.4*	Probable histone deacetylase complex subunit SAP18
*C29F9.5*	Histone acetyltransferase; ortholog of human EP300
*ceh-2*	Homeobox protein; homolog of EMX1/2
*ceh-40*	Homeobox protein; homolog of Exd/Pbx
*cog-1*	Homeobox protein; homolog of Nkx6
*duxl-1*	Homeobox protein; related to dual homeobox Like
*efl-3*	E2F-like (Mammalian transcription factor)
*hda-1*	Histone DeAcetylase 1
*hda-11*	Histone DeAcetylase 11
*hda-3*	Histone DeAcetylase 1
*mxl-2*	Ortholog of human MLX
*mys-4*	MYST family histone acetyltransferase; NuA4 complex
*nfya-1*	DNA binding protein; homolog of human NFYA
*nfyc-1*	DNA binding protein; homolog of human NFYC
*sir-2.3*	NAD-dependent deacetylase
*swsn-5*	SWI/SNF chromatin remodeling complex component
*T26A8.4*	Ortholog of transcription/RNA degradation factor Caf120
*zip-2*	bZIP transcription factor family
*zip-4*	bZIP transcription factor family; related to human CEBPs
*ZK1067.2*	Zinc finger protein; homolog of human ZNFX1

^a^Selected genes from this category, including transcription factors in wTF2.0 [54] and chromatin regulators. All genes are listed in Additional file 3

The hmc-biased genes were enriched with GO biological process terms typically associated with neuronal function (Fig. 4b; Additional file 3). For example, there were 4.0 times more genes with the GO term “synaptic vesicle exocytosis” and 3.6 times more genes with the GO term “calcium mediated signaling” than would be expected for a gene list of this size (FDR < 0.05). Genes with the “synaptic vesicle exocytosis” GO term are particularly suggestive that the hmc has neuronal signaling activity (Table 2; Additional file 3). Also notable in the overrepresented GO terms for hmc-biased genes was “muscle contraction”. Genes within this GO term category include those encoding myosin heavy and light chain proteins, which are associated with muscle function.

**Table 2:** Genes with GO term “synaptic vesicle exocytosis” are enriched in hmc DEGs

Gene	Description
*aex-4*	t-SNARE protein
*cpx-1*	ComPleXin
*pkc-1*	serine/threonine protein kinase; ortholog of PKCe
*ric-4*	Ortholog of human SNAP-25
*snap-29*	SNAP-25 family member
*snt-1*	SyNapTotagmin
*snt-2*	SyNapTotagmin
*snt-3*	SyNapTotagmin
*snt-5*	SyNapTotagmin
*snt-6*	SyNapTotagmin
*syx-2*	SYntaXin
*syx-4*	t-SNARE protein
*tom-1*	TOMosyn
*unc-10*	Rab-3-interacting molecule UNC-10/RIM
*unc-13*	Phorbol ester/diacylglycerol-binding protein UNC-13
*unc-18*	syntaxin chaperone UNC-18
*unc-31*	Pleckstrin; Calcium-dependent secretion activator

To ask if our dataset supports a more neuronal or muscle function for hmcs, we compared our hmc-biased gene set to available expression profiles from isolated cells: 1- isolated larval neurons [31], which we are calling “larval neuron enriched”, and 2- isolated embryonic muscle cells that were analyzed directly or cultured for 24 h to allow the cells to differentiate prior to analysis [32], which we are calling “total muscle enriched” (Table 3, Additional file 4). We found that the hmc cell had more expression in common with both differentiated neurons and muscles (31 and 26%, respectively) than did SGPs (10 and 16%, respectively). One possibility was that hmc had greater overlap because it, like the neurons and muscles, is terminally differentiated, while SGP is undifferentiated. If this were the case, we would expect the overlap of hmc and neurons to be similar to the overlap between hmc and muscles, and these overlapping patterns might represent a “differentiated state” expression pattern. Overall, we found that most of the overlapping genes between hmc and each differentiated cell type were entirely distinct from each other, demonstrating that hmc has specific expression patterns in common with each cell type. We did find one class of genes that was enriched in hmcs, neurons, and muscles (GO term “chemical synaptic transmission”) (Additional file 4); this category includes genes, such as acetylcholine receptors, that are used by both neurons and muscles.

**Table 3:** Overlap between SGP and hmc biased genes and muscle and neuron enriched genes

	SGP biased	hmc biased
Total muscle enriched	16.4% (208/1272)	25.9% (330/1272)
Larval neuron enriched	10.4% (160/1545)	31.2% (482/1545)

### Comparison to SGP enriched genes

Our gene expression analysis differs somewhat from a previous analysis in which the SGP transcriptome was compared to that of all cells of the L1 larva [18]. Kroetz and Zarkower identified 418 genes that were enriched in hermaphrodite SGPs relative to the whole worm. We examined these genes in our dataset and found that 349 of the 418 SGP-enriched genes (83.5%) from their dataset were detected in SGPs in our dataset (mean FPKM > 1). Next, we examined whether these 349 genes found in both datasets were differentially expressed between SGPs and hmcs and found that 293 (84.0%) had higher expression in SGPs than hmcs (Additional file 5). Therefore, many of the SGP-enriched genes defined by Kroetz and Zarkower [18] are also SGP-biased in our dataset.

### Validation of gene expression data

In addition to the SGP-enriched genes identified by Kroetz and Zarkower, the online *C. elegans* database Wormbase (http://www.wormbase.org) annotates 45 protein-coding genes as being expressed in the SGPs and 61 as being expressed in the hmc. We examined these genes in our dataset and found that 35/45 (78%) of the SGP-expressed genes and 52/61 (85%) of the hmc-expressed genes found on Wormbase were detected in our dataset (Additional file 5).

**Fig. 5:**
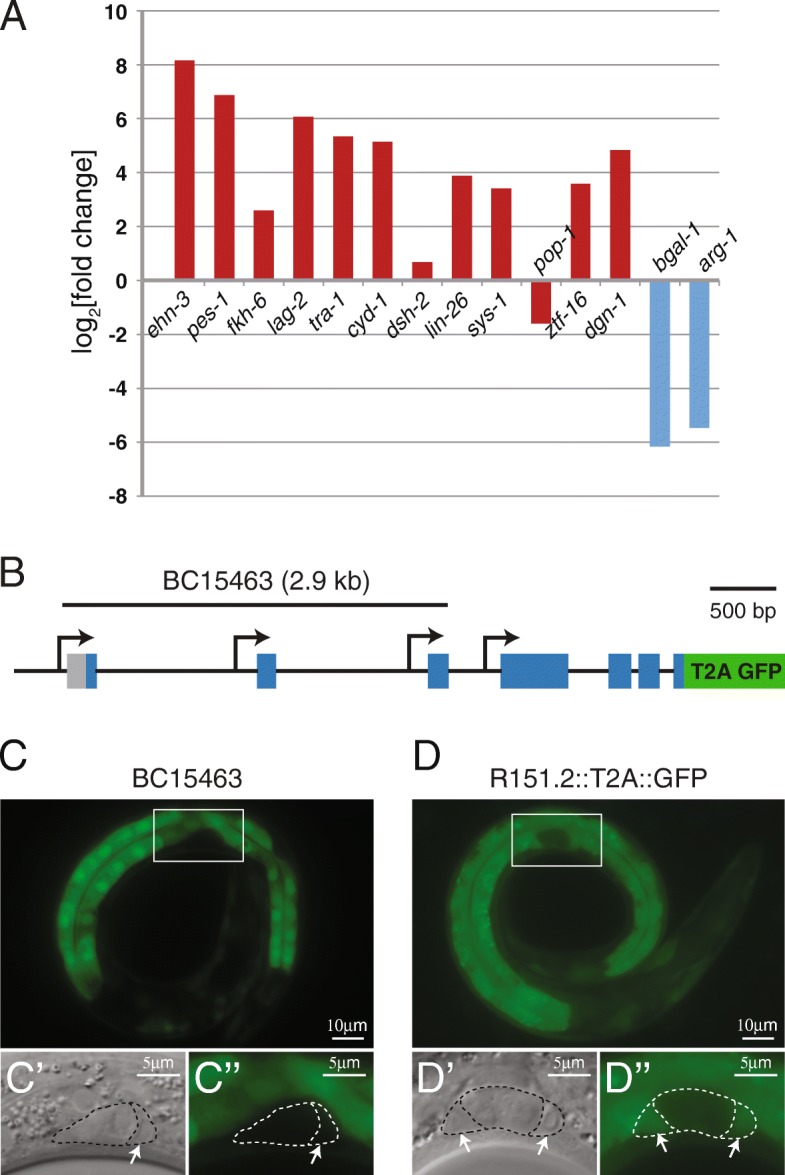
Reporter expression validates differential gene expression. (**a**) Previously published gene reporters show expression of *ehn-3, pes-1 fkh-6, lag-2, tra-1, cyd-1, dsh-2, lin-26, sys-1, pop-1, ztf-16,* and *dgn-1* in SGPs (red) and *bgal-1* and *arg-1* (blue) in hmcs. We detected expression of all of these genes in our dataset (not shown). log_2_[fold-change] in expression between SGPs and hmcs is reported. Positive numbers indicate higher expression in SGPs (red bars); Negative numbers indicate higher expression in hmcs (blue bars). (**b**) The R151.2 locus produces at least eight transcripts from four promoters. The *C. elegans* gene expression consortium generated an R151.2 transcriptional reporter (BC15463). The 2932 bp genomic region used to drive reporter expression in BC15463 is shown; it includes only three of the four known promoters. We created an endogenous R151.2 reporter by using CRISPR/Cas9 to insert the viral T2A peptide upstream of GFP coding sequences and immediately before the R151.2 stop codon. All previously described R151.2 transcripts contain the last exon of the gene; therefore this reporter is predicted to reflect the expression of all R151.2 isoforms. (**c**) The BC15463 reporter is expressed in intestine and cells of the head and tail, but not in SGPs at the L1 larval stage. (**d**) The R151.2::T2A::GFP reporter is expressed in intestine, cells in the head and tail, and in SGPs at the L1 larval stage. (**c-d**) GFP expression is shown for the whole worm (top). DIC (C′-D’) and GFP fluorescence (C“-D”) are shown for higher magnification images of the gonad primordium (bottom). White boxes indicate the area of magnification. Arrows point to SGPs (only one SGP is visible in C′)

The expression of several of these genes has been more thoroughly characterized in direct studies; these include *ehn-3*, *pes-1*, *fkh-6*, *lag-2*, *tra-1*, *cyd-1*, *dsh-2*, *lin-26*, *sys-1*, *pop-1*, *ztf-16*, and *dgn-1* [33–43]. To further assess the quality of our dataset, we examined the expression of these known SGP-expressed genes in our differential gene expression analysis. We found that all of these genes with highly validated expression in SGPs were detected in SGPs (mean FPKM > 1) in our dataset, and all but one of these genes had higher expression in SGPs than hmcs (Fig. 5a). One gene, *dsh-*2, showed only modest enrichment in SGPs, which is consistent with a published reporter for *dsh-*2 showing only weak and inconsistent expression in SGPs [37]. Another of these genes, *pop-1,* was expressed in SGPs (mean FPKM = 4.27), but, in our dataset, had higher expression in hmcs than SGPs. POP-1 protein has been well described to have higher levels of expression in the anterior daughter of many anterior/posterior cell divisions throughout development [44, 45], although post-translational rather than transcriptional regulation has been implicated in this asymmetry. hmcs are the anterior daughters and SGPs are the posterior daughters of MS.appaa and MS.pppaa [2], so the hmcs might be expected to have greater POP-1 protein levels. We found that hmcs have higher levels of *pop-1* transcript, suggesting that transcriptional regulation may be contributing to POP-1 asymmetry in this cell division.

Two genes with well-documented reporter expression in L1 hmcs are *arg-1* [46] and *bgal-1* [13, this work]. We found that both of these genes were expressed in hmcs (mean FPKM > 1) and had higher expression in hmcs than in SGPs (Fig. 5a). Therefore, our dataset contains known SGP- and hmc-expressed genes and our data are consistent with their previously described expression patterns.

As an additional form of validation, we examined strains bearing reporter constructs for genes we found to be highly differentially expressed in L1 SGPs. Of the 10 SGP DEGs with the most significant *p*-values, there were available reporter strains for five (Table 4). We were surprised to find that only two of the five reporters showed detectable expression in SGPs at the L1 stage. One possibility for the lack of detectable fluorescence in SGPs is that expression is below the level of detection using fluorescent reporters. However, two of the genes, R151.2 and *ahcy-1*, had high levels of expression in SGPs (mean FPKM 389.0 and 1606.9, respectively), therefore, it seems unlikely that these genes are below the level of detection with fluorescent reporters. Another possibility is that these gene reporters do not contain all relevant regulatory sequences, and therefore do not faithfully recapitulate the endogenous expression pattern of the gene. For example, the R151.2 locus contains at least eight transcripts that are generated from four different promoters (Fig. 5b). The existing strain that we examined, BC15463, carries an extrachromosomal array in which GFP is driven by 2932 bp of genomic sequence, including only three of the four R151.2 promoters. The BC15463 reporter is expressed in many tissues including intestine, nerve cord, and head and tail neurons, but, notably, is not expressed in SGPs (Fig. 5c). To examine the possibility that the BC15463 reporter construct is missing important regulatory sequences, we generated a novel reporter for R151.2 using CRISPR/Cas9 mediated gene editing [14], to insert GFP at the 3′ end of the intact R151.2 locus. We included a viral 2A peptide upstream of the GFP coding sequence [15] to create a transcriptional gene reporter that should reveal the endogenous expression pattern of the gene and minimize the effect of the fluorescent reporter on the function of the gene (Fig. 5b). Our new R151.2 GFP reporter shows expression in SGPs (Fig. 5d), indicating that at least one of the R151.2 transcripts is expressed in SGPs. We conclude that the BC15463 R151.2 reporter construct does not accurately reflect the complete expression pattern of R151.2.

**Table 4:** Reporter validation of SGP DEGs

gene	log_2_[FC]^a^	p-value*	SGP FPKM	Reporter^b^
*fmo-5*	5.43	2.4E-129	341.7	–
*mrp-2*	4.40	4.5E-75	24.8	**BC12028**
*rpl-30*	3.11	1.7E-73	3359.5	–
*R151.2*	4.92	1.3E-72	389.0	BC15463
*cutl-14*	5.55	4.2E-72	31.4	–
*C31C9.7*	4.94	4.8E-72	178.0	–
*F48G7.10*	4.78	5.8E-69	38.2	**BC11529**
*C30F12.5*	4.37	4.1E-66	94.2	–
*asm-1*	3.94	1.3E-65	18.2	BC10183
*ahcy-1*	4.29	2.1E-65	1606.9	BC11164

*adjusted p-value

^a^ log_2_(fold change)

^b^bold indicates expression in SGPs

Taken together, these analyses validate our gene expression dataset, indicating that we have a robust dataset for examination of gene expression differences between SGPs and hmcs.

## Discussion

In this study, we examined the transcriptomes of two sister cells, one of which is a multipotent progenitor cell (SGP) and the other is a differentiated cell (hmc). We generated a strain of *C. elegans* in which, in the same animals, the SGPs were labeled with a red fluorescent protein, and hmcs were labeled with a green fluorescent protein. We isolated pure populations of SGPs and hmcs from these animals after the SGPs and hmcs had been born, but before the SGPs had further divided, and performed transcriptional analysis on these cells. In total, we identified 5912 genes with differential expression between the two cell types.

SGPs and hmcs are quite transcriptionally distinct, despite sharing a common lineage history. We isolated the cells for analysis approximately 9 h after they were born, but we know that they display different fates much earlier than this. First, hmcs and SGPs migrate in opposite directions almost immediately after their birth [2]. Second, an *enh-3* reporter is expressed in SGPs but not hmcs within 200 min of their birth [34]. Before the cells divide, there is no obvious asymmetry in the mother cell, however, the SGPs are always the posterior daughters of the cell divisions, so it is possible that there is partitioning of differentiation factors within the mother before the cell divides.

Our analysis revealed interesting differences between the expression profiles of the SGPs and their hmc sisters. We found that SGPs express genes that are associated with transcription and translation, as would be expected of a multipotent progenitor that will undergo several rounds of cell division to produce 143 support cells in the hermaphrodite reproductive system. Among the most highly expressed genes in the SGPs are many ribosomal protein components, which would be expected of cells that are poised to undergo proliferative divisions. By contrast, the hmc is a terminally differentiated cell and would not be expected to require significant translational function, and we found that it expresses genes associated with the terminally differentiated fates of both neurons and muscles.

### SGP-expressed transcription factors are likely to include multipotency factors

Pluripotency is distinct from multipotency and is the capacity to generate many different cell types including cells from all three germ layers. In the last decade, much has been learned about the regulation of pluripotency through the study of induced pluripotency in mammalian cells [1], although less is understood about the regulation of multipotency. In mammals, the induction of expression of four core pluripotency factors, OCT3/4, SOX2, KLF4, and MYC, in differentiated cells can convert them into induced pluripotent stem cells (iPSCs) [47, 48]. A slightly different cocktail of human pluripotency factors, including NANOG and LIN28 in place of KLF4 and MYC, was also capable of reprogramming differentiated cells into iPSCs [49]. iPSCs can contribute to all three germ layers when injected into blastocyst embryos, indicating that they are pluripotent. The factors directing pluripotency and multipotency have not been described in worms. We considered the possibility that a mulitpotent state might require some or all of these known mammalian pluripotency factors. In worms, OCT3/4 is encoded by *ceh-6,* SOX2 is encoded by *sox-2,* KLF4 is encoded by *klf-1*, LIN28 is encoded by *lin-28,* and NANOG is not present. We examined *ceh-6, sox-2, klf-4,* and *lin-28* expression in our dataset and found that none of these genes was significantly differentially expressed between SGPs and hmcs (Additional file 5). In worms, MYC is encoded by a gene called *mml-1* (Myc and Mondo-like), which has features of both *Myc* and *Mondo* [50]*.* We found that *mml-1* is expressed at 5.3 times higher levels in SGPs than hmcs (Additional file 5). Therefore, at least five of the six mammalian pluripotency factors do not appear to be important for multipotency in SGPs.

In *C. elegans*, SWI/SNF (SWItching defective/Sucrose Non-Fermenting) chromatin remodeling complexes are important for the multipotency of the SGPs, because mutations in SWI/SNF components cause defects in SGP/hmc cell fate specification [6]. SWI/SNF complexes are also important for the pluripotency of mouse embryonic stem cells [51, 52] and SWI/SNF subunits can facilitate the reprogramming of fibroblast cells into pluripotent stem cells [53]. We favor a model in which SWI/SNF directly controls the expression of multipotency factors. However, it remains possible that there is a general role for chromatin maintenance in cell fate specification, and that the loss of multipotency is an indirect result of the dysregulation of chromatin structure in SWI/SNF mutants. In either case, together, these observations suggest that the mechanisms underlying the maintenance of proliferative potential are likely to be conserved across phyla.

Our goal is to understand the factors that define multipotency, and while the SWI/SNF contribution to multipotency is important, there are clearly additional factors that we have yet to identify. Given that most of the pluripotency factors were not differentially expressed in SGPs, we considered the possibility that SGPs might utilize a different set of transcription factors to establish a multipotent state. The *C. elegans* genome encodes 934 predicted transcription factors [54]. Among the genes with differential expression in SGPs, we identified 175 predicted transcription factor genes (Additional file 5). Thus, we have identified a large number of genes that might be contributing to the regulation of multipotency of SGPs. While we have not yet identified the factors that promote multipotency in the SGPs, some of these SGP-biased transcription factors are good candidates. For example, *efl-3* is known to repress the terminally differentiated fate of apoptosis in the VC ventral motor neuron lineage [55] and may similarly be repressing differentiation to promote multipotency in SGPs. Another interesting candidate is *mxl-2,* which together with *mml-1,* functions as a Myc-like transcriptional activator to regulate cell migration in the male tail [50]. Mammalian MYC is one of the core pluripotency factors, raising the intriguing possibility that a Myc-like transcription factor might work together with a different set of transcription factors to regulate multipotency in *C. elegans*. Additional experiments will be required to determine if these genes are important for multipotency in SGPs.

### Insight into the function of the head mesodermal cell

Almost all of the 959 somatic cells in *C. elegans* have been assigned a biological function, but a striking exception is the hmc cell. While its location and morphology have been carefully described [5, 56, 57], as yet there has been no experimentally derived evidence of its function. The hmc cell occupies a position in the head of the animal and has long processes that lie between the intestine and body wall muscle and run adjacent to the excretory gland, and hmc makes gap junctions with these tissues. These gap junctions perhaps provide a clue to the cell’s function; one suggestion is that hmc may help to coordinate the activity of the muscle in the head and neck of the animal, which may have important developmental roles during the elongation of the embryo [56]. Coordination of the contraction of the muscle surrounding the excretory pore may also be important for excretion. Because the hmc cell lies in the pseudocoelom, and is surrounded by the pseudocoelomic fluid, another possibility is that hmc communicates with surrounding cells using secretory signaling molecules, a suggestion supported by its expression of an extraordinary diversity of innexin forms [58]. However, there are also suggestions that hmc is muscle-like. Its nuclear morphology is more like muscle nuclei than neuronal nuclei [5]. Gene expression studies suggest that at least some expression in hmc is regulated like expression in muscle cells: *hlh-8* is expressed in a subset of muscle cells and hmc, and a region of the *arg-1* promoter that drives expression in vulval and enteric muscles also drives expression in hmc [46].

We compared our hmc-biased genes with those that are enriched in muscles [32] or neurons [31] and found that hmc expresses genes in common with both cell types. Our finding that genes involved in synaptic vesicle exocytosis were enriched in hmc strongly supports the notion that hmc has at least some neuronal-like functions. This point is underscored by the observation that 15 of 23 genes associated with the synaptic vesicle cycle [59] are hmc-biased (Additional file 4) making it highly likely that hmc has some signaling functions. hmc also expresses genes that are characteristic of muscle function, including those encoding components of thick filaments, such as the myosin heavy chain genes *unc-54* and *myo-3* [60]*.* However, hmc-biased genes do not include those encoding thin filament proteins, such as tropomyosin and troponin (Additional file 4), suggesting that hmc does not act as a traditional muscle. In addition, we are unaware of any evidence that hmc contains actin fibers or is contractile in nature. One possibility is that the hmc cell adopts a hybrid fate, with some characteristics of both neurons and muscle.

In mammals, there are a number of cell types that are not neurons but nevertheless use synaptic-like vesicles in regulated exocytosis, including several types of endocrine cells and glia ([reviewed in [61]). For example, pancreatic beta cells use synaptic-like microvesicles (SLMVs) to secrete GABA, which is involved in the regulation of pancreatic endocrine function. If hmc is a secretory cell, we would expect it to manufacture one or more signaling molecules. We therefore looked in our dataset for hints as to what hmc may secrete (Additional File 4). While we have not conducted an exhaustive search, we found that hmc has robust expression of 30 FMRF-like peptides; *flp-1, flp-5, flp-9, flp-10* and *flp-16* are all expressed at very high levels in hmc. Additionally, 11 insulin-related genes are expressed in hmc, including *ins-1* and *ins-17*. Interestingly, hmc also expresses *unc-25*, which encodes a *C. elegans* glutamate decarboxylase, and is required for the synthesis of GABA [62], and *unc-47*, which is required for the packaging of GABA into synaptic vesicles [63], suggesting that, like pancreatic beta cells, hmc may release GABA using SLMVs [64]. Together, these data strongly support a model in which hmc participates in secretory signaling.

### Comparison of this dataset to existing expression information

Recently, Kroetz and Zarkower performed a transcriptional analysis designed to identify genes with higher expression in hermaphrodite SGPs when compared with all cells of the L1 larva, which they called “SGP-enriched” genes [18]. We found that 84% of the SGP-enriched genes were detected and 70% were differentially expressed in SGPs in our dataset. These two RNA-seq experiments would not be expected to identify all of the same genes. For example, because our analysis looks specifically for differential expression between SGPs and hmcs, SGP-enriched genes might not be found in our SGP DEGs if the gene is also expressed in hmc. In addition, the timing of these two gene expression studies was different: we isolated SGPs from newly hatched L1 larvae, while they isolated SGPs from L1 larvae that had been fed and allowed to develop for 9.5 h [18]. This would allow sufficient time for SGPs to begin expressing genes necessary for their development, or in response to feeding, which would not be present in our dataset.

We compared our findings to existing expression information and found that 78% of genes for which SGP expression was reported and 85% of genes for which hmc expression was reported were expressed in the appropriate cell type in our L1 dataset. One reason that annotations on Wormbase might not agree with our dataset is that they do not always include temporal information. The hmc cell is present from embryogenesis through adulthood; and the annotation of hmc expression does not necessarily indicate that the expression is present in the L1 larval stage. SGPs are present in embryos and L1 larvae, so that the timing of expression can also be confounding for genes reported to be expressed in SGPs. For example, the gene *hnd-1* has clear expression in SGPs in embryos, but *hnd-1* expression does not persist into the L1 larval stage [65]. Consistent with this, *hnd-1* did not show appreciable expression in L1 SGPs in our dataset (mean FPKM = 0.04).

Finally, we did a small survey of the publicly available reporters for SGP DEGs with the most significant *p*-values. We found that we could detect expression of GFP in SGPs in only two of the five strains that we examined. To determine if the lack of expression in SGPs was due to incomplete regulatory elements, we generated our own reporter construct for one of the genes, R151.2. We used CRISPR/Cas9 to insert a reporter into the endogenous locus, which should more accurately represent the genuine expression pattern of R151.2. Indeed, consistent with our RNA expression data, we found that with our new construct we were able to detect expression of R151.2 in SGPs. This result strongly supports our mRNA expression analysis results. In addition, we note that considerable caution should be taken when using reporter constructs to exclude expression in particular cell types.

## Conclusions

This work describes the transcriptional profiles of two very different cell types that derive from the same parent cell. One cell, the SGP, is a multipotent progenitor that will undergo multiple divisions to give rise to 143 cells that comprise the complex tissues of the somatic gonad, whereas its sister, hmc, is a terminally differentiated cell of unknown function. These sister cells are transcriptionally quite different; we identified almost 6000 genes that were differentially expressed between these two populations of cells. Pathway enrichment analysis revealed that the SGP-biased genes are enriched with those that function in transcription and translation. More specifically, we identified 175 genes that encode transcription factors that were more highly expressed in SGP relative to hmc. These transcriptional regulators provide excellent candidates for studies of the factors underlying multipotency. Interestingly, we observed that the hmc cell, which has not yet been functionally characterized, expresses genes that are consistent with both neural and muscular functions.

## Additional files

Additional file 1: **Table S1.** Primers used in this study. **Table S2.** Quality control metrics for RNA-sequencing libraries. (XLSX 10 kb)

Additional file 2: Differential gene expression analysis results. Included in this file are: 1- all detected transcripts (mean FPKM ≥1 in at least one cell type), 2- SGP-biased DEGs (*p* ≤ 0.01 and fold-change ≥2), and 3- hmc-biased DEGs (p ≤ 0.01 and fold-change ≥2). (XLSX 2025 kb)

Additional File 3: GO term enrichment analysis of the differentially expressed genes. Included in this file are: 1- GO-slim Biological Process analysis of SGP DEGs (FDR < 0.05), 2- SGP-biased genes with the GO term [GO:0006366] “transcription from RNA polymerase II promoter.”, 3- GO-slim Biological Process analysis of hmc DEGs (FDR < 0.05), and 4- hmc-biased genes with the GO term [GO:0016079] “Synaptic vesicle exocytosis.” (XLSX 33 kb)

Additional File 4: Comparisons of this dataset to muscle and neural expression datasets. Included in this file are: 1- total muscle enriched genes [32] that are also SGP-biased or hmc-biased, 2- larval pan-neural enriched genes [31] that are also SGP-biased or hmc-biased, 3- genes that are expressed in muscle, neuron, and hmc, 4- GO terms for genes that are expressed in muscle and hmc. 5- GO terms for genes that are expressed in neuron and hmc. 6- genes that are involved in the synaptic vesicle cycle [59], 7- genes that encode components of thin and thick filaments of body wall muscle [60], and 8- genes that encode FMRF-like and insulin-like peptides. (XLSX 159 kb)

Additional File 5 Comparisons of this dataset to other publicly available datasets. Included in this file are: 1- SGP-biased genes that are also annotated as expressed in SGPs on http://wormbase.org, 2- hmc-biased genes that are also annotated as expressed in hmcs on wormbase.org, 3- SGP enriched genes [18] that are also detected in our study, 4- *C. elegans* transcription factors in the wTF2.0 dataset [54] that are SGP-biased in our dataset, and 5- expression results for *C. elegans* homologs of pluripotency factors. (XLSX 152 kb)

## Data Availability

The RNA sequencing dataset generated during the current study is available in the NCBI SRA repository, accession number PRJNA506274, and the results are included with this article in tables and additional files.

## Abbreviations

AC: Anchor cell
DEG: Differentially expressed gene
DTC: Distal tip cell
FACS: Fluorescence-activated cell sorting
FDR: False discovery rate
FPKM: Fragments Per Kilobase of exon per Million fragments mapped
GFP: Green fluorescent protein
GO: Gene ontology
hmc: head mesodermal cell
iPSC: induced pluripotent stem cell
L1: first larval stage
PCR: Polymerase chain reaction
PGC: Primordial germ cell
SGP: Somatic gonadal precursor cell
SWI/SNF: SWItching defective/Sucrose Non-Fermenting chromatin remodeling complex
